# Analysis of longitudinal oncology quality of life (QoL) data - are we getting it right?

**DOI:** 10.1186/1745-6215-14-S1-P105

**Published:** 2013-11-29

**Authors:** Kim Cocks, Puvan Tharmanathan, Adam Smith

**Affiliations:** 1 York Clinical Trials Unit, Department of Health Sciences, University of York, York, UK; 2 York Health Economics Consortium (YHEC) and Research Innovation Office, University of York, York, UK

## Background

Quality of Life (QoL) data from oncology trials may have missing data which cannot be assumed to be missing completely at random (MCAR) [1]. Ignoring this missing data in analysis may introduce bias. A number of statistical techniques to deal with informative missing data are available [2], but may be underutilised.

## Methods

We searched MEDLINE (2002-2012) to identify oncology trials reporting longitudinal analysis of QOL data. The appropriateness of the analysis was reviewed and trials reporting QOL as primary/secondary endpoint were assessed for reporting quality using the CONSORT extension for PROs [3].

## Results

69 RCTs reporting longitudinal QOL analyses were identified. 29 (42%) use an analysis to account for the nature of the missing data. Methods varied widely, eg pattern-mixture models, conditional linear models, QTWiST, joint longitudinal models, generalised estimating equations, selection models and Markov models. Fourteen papers used more than one method check the robustness of their results.

## Conclusions

In order for QOL data to adequately inform clinical decision-making the correct analysis needs to be performed. Statistical methods ignoring the missing data were found to over-estimate QOL but it was rare for the significance of QOL differences between treatments to change. A strategy for appropriate analysis of QOL data will be presented using case studies to highlight where ignoring informative missing data could alter the conclusions regarding treatment differences.
